# Creating a safety net for patients with depression in primary care; a qualitative study of care managers’ experiences

**DOI:** 10.1080/02813432.2018.1529018

**Published:** 2018-10-12

**Authors:** Irene Svenningsson, Camilla Udo, Jeanette Westman, Shabnam Nejati, Dominique Hange, Cecilia Björkelund, Eva-Lisa Petersson

**Affiliations:** a Department of Primary Health Care, Institute of Medicine, The Sahlgrenska Academy, University of Gothenburg, Gothenburg, Sweden;; b Primary Health Care, Närhälsan Research and Development, Region Västra Götaland, Sweden;; c School of Education, Health and Social Studies, Dalarna University, Falun, Sweden;; d Centre for Clinical Research, Dalarna, Sweden;; e Division of Family Medicine, Department of Neurobiology, Care Sciences and Society, Karolinska Institute, Stockholm, Sweden

**Keywords:** Care manager, Depression, Healthcare professionals, Primary health care, Qualitative study

## Abstract

**Objective:** The aim of this study was to explore nurses’ experiences and perceptions of working as care managers at primary care centers.

**Design:** Qualitative, focus group study. Systematic text condensation was used to analyze the data.

**Setting:** Primary health care in the region of Västra Götaland and region of Dalarna in Sweden.

**Subjects:** Eight nurses were trained during three days including treatment of depression and how to work as care managers. The training was followed by continuous support.

**Main outcome measures:** The nurses’ experiences and perceptions of working as care managers at primary care centers.

**Results:** The care managers described their role as providing additional support to the already existing care at the primary care center, working in teams with a person-centered focus, where they were given the opportunity to follow, support, and constitute a safety net for patients with depression. Further, they perceived that the care manager increased continuity and accessibility to primary care for patients with depression.

**Conclusion:** The nurses perceived that working as care managers enabled them to follow and support patients with depression and to maintain close contact during the illness. The care manager function helped to provide continuity in care which is a main task of primary health care.

**Key Points**
  The care managers described their role as an additional support to the already existing care at the primary care center.
 •  They emphasized that as care managers, they had a person-centered focus and constituted a safety net for patients with depression.
 •  Their role as care managers enabled them to follow and support patients with depression over time, which made their work more meaningful.
 •  Care managers helped to achieve continuity and accessibility to primary health care for patients with depression.

## Introduction

The number of people with depression is increasing worldwide [1], affecting people in all ages. Almost 1 million lives are lost to suicide due to depression worldwide each year [1]. In Sweden, the life time risk of depression is around 35 percent for women and 25 percent for men [2]. Depression is associated with a large burden of disease and serious consequences, such as decreased quality of life and reduced ability to cope with daily life [1,3], and increased risk of disease [4]. The main symptoms of depression are low mood and/or loss of interest combined with feeling sad, sleep disturbance, weight change, impaired concentration, feelings of hopelessness and fatigue. A depressive episode is categorized as mild, moderate, or severe on the basis of the symptoms and the number of previous episodes [1,3]. The prevalence of major depression is reported to be 12 percent in the Swedish primary care registry, and 80 percent of people with major depression are treated solely within primary health care (PHC) [3,5]. Since most people with depressive symptoms are treated within PHC [3,5,6], strengthening primary health care for patients with depression is an urgent priority. The comprehensive approach through implementing a care manager in a collaborative care organization in PHC, i.e. a professional specifically trained for managing and coordinating the care for the patient with depression, has positive effects [7]. Studies showed that after introducing care managers, the prescribing of antidepressants became more adequate in addition to patients reporting reduced symptom burden [7]. The use of care managers was also cost effective [8–10]. These positive effects, on both the individual and organizational levels, motivate testing the use of a care manager function on a large scale.

The primary health care system plays an important role in providing adequate care for patients with depression [6]. The current treatment options are visiting a General Practitioner (GP), combined with antidepressant medication and/or psychotherapy [3]. Some groups have lower medical adherence with drugs than others [11], where support given by mental health nurses has shown no effect of antidepressant prescription [12]. Because of inadequate support such as limited follow-ups, many patients with depression discontinue antidepressants during the first weeks of treatment, with increased risk of relapse [6,13,14].

Previous studies from the US and UK have shown that organizational changes, such as implementing a care manager function in a collaborative care organization that provides individual support to patients with depression who are treated at primary care centers (PCC)s, lead to faster recovery and return to work [8–10]. Primary health care is organized differently in different countries, and the use of care managers has not yet been scientifically tested in Swedish primary health care. It is thus important to carry out studies to evaluate whether using care managers has the same effects in Sweden as in other countries [15]. This study is a part of a larger Care Manager Intervention study, PRIM-CARE, which is described elsewhere [16].

In the present study we investigated how care managers work in Swedish primary care.

## Aim

The aim of this study was to explore nurses’ experiences and perceptions of working as care managers in primary care health centres.

## Methods

### Design

This qualitative study used focus group discussions [17,18]. Data were analyzed with Systematic Text Condensation [19].

### The Care manager

In the Care Manager project described in our study, the care managers were nurses who provide support for patients with mild to moderate depression. The nurses worked as care managers as a part of their work as nurses at the PCCs. The estimated amount of time was allotted 25 percent at a PCC with ∼10,000 listed people. Nurses at PCCs provide advice and nursing based on a health perspective. In the care manager function, the continuity of relationship was in focus. The purpose of the training was to gain in-depth knowledge of about collaborative care and treatment of depression. After the patient´s first appointment with the GP, the care manager contacted the patient regularly. The first contact was a face-to-face meeting. This meeting was followed by six telephone calls over the three months after the patient has been diagnosed with depression. Via the face-to-face meeting and telephone calls, the care manager followed the patient´s symptoms, provided information and support regarding medical and psychological treatment options and encouraged the patient to engage in self-care for example, behavioural activation, advice about food, medication, and sleeping problems. The contact between the care manager and the patient was structured, and the care manager and patient agreed in advance when each contact would take place, and a sort of contract, documenting agreed upon goals and care planning, was established using a person-centered approach [20]. The person-centered approach involved seeing the patient as an equal partner and an expert, putting the person at the center of decisions. Each time the care manager contacted the patient, the patient used a short depression self-assessment instrument MADRS-S [21,22] to measure the depth of his or her depression, and the care manager used the result as a basis for support to the patient and discussing self-care. The MADRS-S rating was important for assessing the course of the depression and continued treatment, for example drug use, with particular focus on suicidal risk. MADRS-S has been used as the patient’s instrument in depression care with good results [23]. The contacts between patient and care manager occur over a longer period. The care manager cooperates closely with the patient’s GP and with other members in the care team.

### Setting and participants

Twenty-three PCCs at different sites in two counties in Sweden, both urban and rural areas, were included in the randomized controlled trial PRIM-CARE. Twelve of the PCCs were randomized to a control group, and eleven to an intervention group. A care manager program was implemented at the centers included in the intervention group, and one nurse was trained as a care manager. These eleven nurses, were invited to focus group discussions. The participants were informed that the contents of the discussion would be kept confidential, that the discussion would be audio-recorded and transcribed verbatim, and that participation was voluntary and they were free to stop at any time.

### Data collection

Three focus group discussions were conducted and moderated by IS and ELP with CU and SN as assessors, to support the moderators in the discussions. The participants were asked to discuss their experiences of working as care managers. Three nurses could not participate because of lack of time, leaving eight nurses to be included. The nurses were divided into two groups: group one, *n* = 4 and group two, *n* = 4. These two groups were conducted two months after the intervention had started. Six months after the intervention started, the care managers in group one and two met and participated in a third focus group, *n* = 6. The informants had worked as nurses for between 5 and 35 years. All were RNs, and some were districts nurses and psychiatric nurses. IS and SN are district nurses; ELP is an occupational therapist and CU is a social worker. IS, ELP, and CU have doctoral degrees with experience from primary health care and psychiatry. Each focus group discussion was electronically recorded and lasted about one hour. The focus groups took place at a conference center in Gothenburg. The questions explored in the focus groups were: What is it like to work as a care manager? How do you experience your role as a care manager at the PCC? What role does the care manager have at the PCC?

### Analysis

The data analysis was performed in collaboration between IS, CU, and ELP using systematic text condensation [19]. Analysis was performed according to the following steps: 1) Initially, all the transcribed interviews were read to obtain an overall impression, keeping an open mind and keen awareness to the participants' voices. 2) Meaning units were identified, representing different aspects of participants’ experiences of working as care managers, and coded accordingly. 3) The contents of each coded group were condensed and sorted into a few subgroups. By reviewing meaning units within the subgroup, we reduced the content into a condensate – an artificial quotation maintaining the original terminology applied by the participants. 4) Finally we summarized the contents of each code group as general descriptions and concepts [19]. When summarizing the contents in each sequence, it is easy to see when the conclusion emerges and the condensates, which come from empirical data, present coherent stories [19].

## Results

The participants willingly shared their experiences of working as care managers in primary health care. They perceived the work as meaningful because of being able to provide patients with enhanced support, such as better continuity and increased accessibility of care. They described care managers as coordinators of care. Five categories emerged from the analysis: Coordinating care, Working together, Empowered or powerless, Providing person-centered care, and Following and supporting the patient. The findings are elaborated upon below, illustrated by selected quotations.

### Coordinating care

Participants described their work as a care manager as being a contact person who was available for the patient over time. They saw themselves as a ‘spider in the web’, i.e. a person who had an overview of the patient’s health care contacts, who was an extra link to the GP, and who could coordinate or hasten the care process. They encouraged and guided the patient, and gave feedback to the patient and the GP.

For me, being a care manager means that the patient has someone to turn to, that they have this contact person, like an old-time district nurse. So this function, `the spider in the web´, benefits the patients tremendously. (Focus group (FG) 1)

As a care manager, they represented the patient within and outside the primary care center. For example, when a patient waited for contact with a therapist, the care manager could try to bring about an earlier appointment if needed. Participants also saw themselves as a bridge between the patient and various health care contacts. As care managers, they supported the patient to initiate and keep contact with formal and informal support networks. Informal networks, such as friends and family, were regarded as important for the patient’s recovery in the long term.

I think the care manager role is much about helping the patient become aware of what is available. Putting the patient in the center and looking at different functions that might help them to sustain their recovery. I’m kind of a, well … coordinator, who can help with various contacts, both in terms of health care and other social functions… To provide guidance… (FG 2)

### Working together

Although psychotherapists were mentioned a few times, working together mainly involved working and having a more open communication with the GP. Increased cooperation between the care manager and the GP improved patient care, e.g. when the patient could more easily get a response to a brief question from the GP through the care manager. In addition, it was also supportive for themselves in their professional role. The participants experienced the increased cooperation with the GP as having the potential to unburden both the GPs and themselves and contributed to shared responsibility.

There we were, all three of us, working together, and consequently sharing responsibility. It has become a shortcut in some ways. The cooperation between different professions is very important since the work becomes less divided between RNs and GPs. We are working together in a completely different way than before. (FG3)

Open communication with different professions was important for the care manager for optimum functioning, especially between the GPs and the care manager. Many of the participants had experienced that some of the GPs, especially the younger ones, initiated a closer cooperation with them as care managers than before entering this new role.

### Empowered or powerless

The work as a care manager was by many of the participants experienced as empowering for themselves in their professional role. For example, as care manager, they felt empowered to make independent decisions that they perceived were in favour of the patient, e.g. giving a patient a quick GP appointment despite many patients in line.

I have taken the power to give them appointments to the same doctor although I’m not really allowed to do that … When there are few doctors’ appointments left there are certain rules how to book the patient, but I 've gone a bit beyond that and no one has said anything. My goal is for the patient to see the same doctor next time. (FG 3)

The participants also described that sometimes they made an independent assessment about the patient’s situation and emotional status, and then discussed this with the GP. However, one difficult part in the care manager role was when the GPs failed to offer patients contact with the care manager. This was due to the GP’s lack of knowledge regarding the existence of the new function, or the GP being stuck in old working routines. In these situations, the participants experienced lacking the authority and power to influence the GPs.

It is the young and new doctors who are interested, remember and see the value of this. I would like to get more feedback from the older doctors… I feel like they just continue working in old routines. I think that the elderly doctors are so bound to what they have always done and feel they will soon retire so they do not take in anything new, although the patients would gain from the function of care manager, but are never offered it… (FG1)

### Providing person-centered care

To make the patient feel acknowledged, listened to and not felt left alone were important parts of the care manager role, according to the participants. The recurrent scheduled telephone calls enabled this person-centered focus. Further, the telephone calls facilitated building a relationship with the patient and made it possible for them to provide the patient with individualised support.

It feels like it’s a good relationship. They can turn to me and they do not have to tell their story to so many people. I actually get to know the person, not merely the situation she or he is in right now. (FG1)

The participants’ experiences of working as care manager meant being more involved in the patient’s situation as a whole. This in turn contributed to feelings of meaningfulness and importance for them in their role as care manager. In addition, every scheduled telephone call with the patient was initiated by filling out an assessment instrument (MADRS-S). The assessment instrument was described as helping both the care manager and the patient to discover also small nuances of recovery, which could then be discussed further between the patient and the care manager.

I thought we got good contact during the first call and we got even better contact when I called the second time. It felt natural to me and, well, he felt better, so it was positive. It felt meaningful to me… I see this as quite an important role. I feel like I'm significant. (FG2)

The care managers considered that the combination between the assessment instrument and the dialogue was helpful in establishing communication and enabling patient-centered care. For example, the dialogues could continue from the results found in the assessment instrument and could also deal with medical treatment or physical activities. The care manager also gave the patient the opportunity to ask questions.

### Following and supporting the patient

The care managers felt they provided a safety net for the patients that they experienced had been lacking before the existence of care managers. The participants perceived they provided continuity and availability in the contact with the patient. In addition, the follow-up was structured in terms of recurrent scheduled telephone calls and the use of the MADRS-S assessment instrument. Patients were therefore not forgotten or left to cope on their own in their depression by the professionals.

The keywords are immediate caretaking and follow-up so the patient is seen and acknowledged. (FG1)

The participants perceived that the recurrent telephone calls and the assessment instrument enabled a trusting relationship with the patient. The continuity also helped the care manager to follow the patient’s process as a whole, which prevented a patient from `getting lost/falling between contacts´. Because of the extra support, continuity, and availability, the care managers perceived that they constituted a safety net. They said they could also follow patients who were not so demanding and could immediately catch them if they became worse.

I catch patients who are usually quiet and not so demanding. To a great extent I see myself as someone who follows up and tries to prevent that any patient of mine falls through… (FG 3)

The participants thought they were more available for patients than before starting to work as care managers. The patients could easily contact the care manager if they felt a need to. Despite this possibility, the patients almost never did contact the care managers. According to the participants, this was probably due to the scheduled telephone calls.

The patients know that they can ask me questions and I’ll try to find the answer and get back to them. This means that there is a continuity which I believe means a lot. (FG 1)

The participants felt that as care manager they could build a trusting relationship with the patient. The participants believed that this trusting relationship helped the patients recover more quickly -than if there was no contact with a care manager.

## Discussion

### Main results

The care managers described their role as an additional support to the already existing care at the PCCs. They emphasized that as care managers they had a person-centered focus and constituted a safety net for patients with depression. They felt empowered to make independent decisions and had the opportunity to hasten care in response to the patient's individual needs, and they worked closely together with other members in the health care team. However, they described feeling frustrated when they lacked the authority to convince GPs to inform patients about the role of care managers and to offer to put the patient in touch with a care manager. As care managers, they scheduled regularly telephone contacts with the patient that enabled early discovery of possible deterioration. In addition, this study showed that when the patient had the opportunity to easily contact the care professionals, they seldom used this opportunity.

Results in this study also indicated that the use of MADRS-S in the structured and systematic telephone follow-ups made it possible to identify person-centered topics, to involve the patient in the care process, and to increase the patient’s understanding of their depression and its consequences. The participants perceived that the care manager helped achieve one of the main tasks of primary health care; i.e. increased continuity and accessibility of primary health care for patients with depression.

### Strengths and weakness

This is the first study to explore what it is like to work as a care manager for patients with depression who are treated in Swedish primary health care. The care manager program was based on methods used in international studies that are described in manuals for care managers [7,16]. In this study, the care managers received regular guidance from supervisors through personal meetings or telephone calls. However, the study also has several limitations that must be considered. Firstly, the care manager model was developed recently, and the results reflect the experiences of working as a care manager within the structure of a short-term, randomized, controlled trial. If the care managers had worked as care managers for a longer time, the results might have been more extensive. It is also possible that the method will evolve over time and perhaps may become more adapted to the needs of the unique circumstances of each PCC. Secondly, the discussions were conducted in groups, which may have affected the participants' responses. Thirdly, the discussions were conducted by IS and ELP with CU and SN as assessors, and all four were also involved in the intervention. This may have influenced the participants such that they avoided discussing the negative aspects of being a care manager. A diverse sample of different statements is important for the findings to be valid. The participants did describe many different situations of their work as a care manager with varying outcome. Thus, we believe the complexity to be sufficient to show a relatively good picture of the care manager’s work.

### Findings related to other studies

Care managers can provide systematic and regular support to patients with depression. Previous studies have found that such support reduces the risk of interrupted treatment, speeds up the recovery process, and reduces the risk of relapse [24]. Those results are in line with the perceptions of the care managers in our study. The person-centered approach in the regular contacts, with individualized discussions and individually adapted support in everyday life, provided the patients with the knowledge and tools to manage the disease, which has previously been found important [25]. The results in our study also support previous results showing that health care professionals want to engage the patient in care [24].

At its best, teamwork improves communication and enables the team to manage complex cases, enhancing patient care [26]. The care managers in our study also experienced this. The current study indicated an improved collaboration when the members of different professions worked together, especially when cooperation between care managers and GPs worked well. These findings are in line with the results of a previous study [27] and ensures that the care chain is held together on an inter- and intra-organizational level. Formalized collaboration between team members and the provision of support by the organization are important [28], since strong leadership and engaged GPs are crucial factors when implementing a care manager organization [27].

The care manager program gives care managers the opportunity to make independent decisions, which the care managers in our study perceived as being in line with patients’ preferences. When nurses are able to influence their own work situation and feel empowered to make independent decisions, it contributes to feelings of meaning, self-efficacy, and satisfaction, in addition to increasing the effectiveness within the organization [29]. On the other hand, our study showed that when the GPs failed to remember that they should send the patients to care managers, the care managers experienced feelings of powerlessness and frustration over the fact that some patients were not given the opportunity to have a care manager. A previous study has shown that health care professionals do not always engage in reforms even if there are positive aspects from the patients’ perspective [30]. According to our study, scheduling telephone calls and using MADRS-S in the patient dialogues provided the opportunity to build a trusting relationship with the patient, and the care managers perceived that the trusting relationship helped the patient to better manage their depression. This type of shift from didactics to encouragement has earlier been shown to empower the patient and to improve quality of care as a result [28]. Furthermore, the care managers’ ability to follow and support the patient over time contributed to the building of a relationship and enabled a person-centered care. Better relationships and more personalized care may, in turn, lead to improvement in both physical and psychological health status [31]. The quality of the relationship between the professional and the patient has a major impact on the care outcome [32].

To gain more knowledge about care management in Swedish PHC, there are also ongoing studies of patients’ and physicians’ perspectives within the larger PRIM-CARE Intervention study.

## Conclusion

The results in this study show that having a care manager function makes it possible to follow up and support patients with depression. In addition, it provides a safety net for patients. According to the results in our study, care managers perceive that they improve care for patients with depression and help to provide continuity and accessibility which make their work more meaningful and empowering.
